# Juvenile delinquency, welfare, justice and therapeutic interventions: a global perspective

**DOI:** 10.1192/pb.bp.115.052274

**Published:** 2017-02

**Authors:** Susan Young, Ben Greer, Richard Church

**Affiliations:** 1 Imperial College London, London, UK; 2 Broadmoor Hospital, Crowthorne, UK; 3 South London and Maudsley NHS Foundation Trust, London, UK

## Abstract

This review considers juvenile delinquency and justice from an international perspective. Youth crime is a growing concern. Many young offenders are also victims with complex needs, leading to a public health approach that requires a balance of welfare and justice models. However, around the world there are variable and inadequate legal frameworks and a lack of a specialist workforce. The UK and other high-income countries worldwide have established forensic child and adolescent psychiatry, a multifaceted discipline incorporating legal, psychiatric and developmental fields. Its adoption of an evidence-based therapeutic intervention philosophy has been associated with greater reductions in recidivism compared with punitive approaches prevalent in some countries worldwide, and it is therefore a superior approach to dealing with the problem of juvenile delinquency.

Recent years have seen sustained public and academic interest in criminality and mental health, with attention often focused on antisocial behaviour by children and adolescents. The scale of the problem of juvenile delinquency has provoked mixed responses from governments and the media across the world, with calls for improved rehabilitation and support for juvenile offenders competing with voices advocating more punitive approaches.^1^ Meanwhile, decades of rigorous academic scrutiny have shed light on the complex and diverse needs of children who come into conflict with the law.^2–5^ Much of the growing body of literature on juvenile offenders shows considerable overlap between criminological, social and biomedical research, with a consensus emerging around the significance of a developmental understanding of the emergence of juvenile delinquency.

Importantly, juvenile offenders have consistently been identified as a population that suffers from a markedly elevated prevalence and severity of mental disorder compared with the general juvenile population.^6,7^ Meeting the needs of these young offenders presents practical and ethical challenges concerning treatment and management, including liaison with other agencies.

## What is juvenile delinquency?

### Who counts as juvenile?

Juvenile delinquency is a term commonly used in academic literature for referring to a young person who has committed a criminal offence, although its precise definition can vary according to the local jurisdiction. The specific reasons underlying these differences are unclear, but they may arise from the lack of an agreed international standard.^8^

A ‘juvenile’ in this context refers to an individual who is legally able to commit a criminal offence owing to being over the minimum age of criminal responsibility, but who is under the age of criminal majority, when a person is legally considered an adult. The minimum age of criminal responsibility varies internationally between 6 and 18 years, but the age of criminal majority is usually 18 years.

In some cases individuals older than 18 years may be heard in a juvenile court, and therefore will still be considered juveniles; indeed, the United Nations (UN) defines ‘youth’ as between 15 and 24 years of age. The term ‘child delinquents’ has been used in reference to children below the age of 13 who have committed a delinquent act,^9^ although elsewhere ‘children’ are often defined as being under 18 years of age. The term ‘young offenders’ is broad, and can refer to offenders aged under 18 years or include young adults up to their mid-20s.

### What is a crime?

A ‘delinquent’ is an individual who has committed a criminal offence. Delinquency therefore encompasses an enormous range of behaviours which are subject to legislation differing from one jurisdiction to another, and are subject to changes in law over time. Whereas acts of theft and serious interpersonal violence are commonly considered to constitute criminal offences, other acts including alcohol consumption and sexual behaviour in young people are tolerated to very differing degrees across the world. Sometimes these differences arise as a consequence of historical or cultural factors, and they may be underpinned by traditional religious laws, such as in some Middle Eastern countries. Some offences may be shared between jurisdictions but be enforced to differing standards – for instance, ‘unlawful assembly’, often used to prevent riots, is applied in Singapore to young people meeting in public in groups of five or more as part of police efforts to tackle youth gangs. Furthermore, ‘status offences’ – acts that would be permissible in adults but criminalised in children, such as consumption of alcohol or truancy – not only vary between jurisdictions, but contribute to discontinuity when comparing juvenile delinquency with adult populations in the same jurisdiction.

Lack of clarity can also arise in jurisdictions where a young offender is processed via a welfare system rather than a youth justice process. Countries with a high minimum age of criminal responsibility may not technically criminalise young people for behaviour that would normally be prosecuted and therefore classed as ‘delinquent’ elsewhere.

Not all incarcerated juveniles are ‘delinquent’, since some may be detained pre-trial and may not be convicted of an offence. Even if convicted, it would be wrong to assume that every ‘juvenile delinquent’ meets criteria for a diagnosis of conduct disorder; offences vary considerably and may not be associated with a broad repertoire of offending behaviour. Also, most ‘juvenile delinquents’ do not pose an immediate risk of violence to others, and the vast majority of convicted juveniles serve their sentences in the community.

To meet the diagnostic criteria of conduct disorder requires evidence of a persistent pattern of dissocial or aggressive conduct, such that it defies age-appropriate social expectations. Behaviours may include cruelty to people or animals, truancy, frequent and severe temper tantrums, excessive fighting or bullying and fire-setting; diagnosis of conduct disorder can be made in the marked presence of one of these behaviours.^10^

Overall, the term ‘juvenile delinquent’ is used extensively in academic literature, but requires some care. It can be a potentially problematic term, and in some contexts can strike a pejorative tone with misleading negative assumptions. For several years the UN has used the phrase ‘children in conflict with the law’ to describe the breadth of the heterogeneous group of individuals under the age of 18 who have broken the law or are at risk of doing so.

## General principles of juvenile justice

### Welfare *v.* justice models

The sentencing of an individual convicted of a criminal offence is largely driven by three key considerations: retribution (punishment), deterrence and rehabilitation. In the case of juvenile offenders the principle of rehabilitation is often assigned the greatest weight.^11^

Special consideration for juveniles within the criminal justice system is not a new concept. In Roman law, the principle of *doli incapax* protected young children from prosecution owing to the presumption of a lack of capacity and understanding required to be guilty of a criminal offence. Most countries have some provision for special treatment of children who come into conflict with the law, however, the degree to which this is provided varies across the world.^1,12^ In some countries a ‘welfare’ model prevails, which focuses on the needs of the child, diagnosis, treatment and more informal procedures, whereas other countries favour a ‘justice’ model, which emphasises accountability, punishment and procedural formality.

Belgium is frequently cited as an example of a country with a strong welfare process, supported by a high minimum age of criminal responsibility of 18 years. Similarly, France built a strong welfare reputation by placing education and rehabilitation at the centre of youth justice reforms in the 1940s. New Zealand in 1989 established the widely praised system of Family Group Conferencing as an integral part of youth justice, with a focus on restoration of relationships and reduction of incarceration that would be considered part of a welfare approach. In contrast, the UK and the USA have traditionally been associated with a justice model and low age of criminal responsibility – 10 years in England and Wales, and as low as 6 years in several US states.

Within welfare or justice models, a young person may at some point be ‘deprived of liberty’ – defined as any form of detention under official authorities in a public or private location which the child is not permitted to leave. Locations in which children may be deprived of liberty include police stations, detention centres, juvenile or adult prisons, secure remand homes, work or boot camps, penitentiary colonies, locked specialised schools, educational or rehabilitation establishments, military camps and prisons, immigration detention centres, secure youth hostels and hospitals.^13^

### Between the less and more punitive systems

The UN supports the development of specialised systems for managing children in conflict with the law. When the first children's courts were set up in the USA in the 1930s, they were widely praised as a progressive system for serving the best interests of the child. Although informality was championed as a particular benefit, in the 1960s substantial concerns arose about due process and the protection of the legal rights of minors. The subsequent development of formal juvenile courts occurred in the context of a continuing ethos of rehabilitation of young people, with a move away from incarceration of juveniles in the 1970s, especially in Massachusetts and California. However, following a marked peak in juvenile offending statistics during the 1980s and 1990s, public and political opinion swung firmly in a more punitive direction. This was accompanied by legal reforms that increased the severity of penalties available to juvenile courts and lowered the age threshold for juveniles to be tried in adult criminal courts.

When the UN Convention on the Rights of the Child entered into force in 1990, the USA was not a signatory owing to 22 states permitting capital punishment of individuals who had committed their crimes as juveniles. It is reported that 19 juvenile offenders were executed in the USA between 1990 and 2005. Although this number may represent a small percentage of the total who faced the death penalty in the USA during that period, the practice was widely criticised by international bodies and organisations.^14^ A landmark ruling in the US Supreme Court^15^ outlawed the execution of juvenile offenders in the USA, but to date a small number of countries worldwide still implement this practice, sometimes as a result of religious laws.

However, it would be wrong to assume that welfare systems are automatically preferable to a juvenile justice approach, since welfare arrangements can be equally coercive in terms of deprivation of liberty of juveniles. They may lack due process, safeguards for obtaining reliable evidence from young people, processes for testing evidence, and procedures for scrutiny or appeal following disposal.

## Trends in youth crime

The USA witnessed a dramatic increase in arrest rates of young people for homicide and other violent crimes in the 1980s and 1990s, sometimes referred to as the ‘violence epidemic’.^16^ The ensuing moral panic led to harsh and punitive policy changes in juvenile justice and, although official statistics document a subsequent fall of 20% in court case-loads between 1997 and 2009, victimisation surveys have indicated a degree of continuity in high levels of offending, consistent with a reported increase in juvenile offending between 2000 and 2006.^17^

In common with the USA and several other high-income countries, the UK also experienced a rise in juvenile offending in the 1980s and 1990s, but figures from the Youth Justice Board for England and Wales appear to indicate a general improvement in recent years. Between 2009/2010 and 2014/2015 a 67% reduction has been observed in the number of young people entering the juvenile justice system for the first time, a 65% reduction in the number of young people receiving a caution or court disposal and a 57% reduction in the number of young people in custody.^18^ These figures support an overall decrease in juvenile offending noted since the early 1990s.^19^

Youth crime figures from Australia have documented a 4% reduction in the overall number of young offenders in 2013/2014,^20^ although the number of violent offences committed by young people in the urbanised and densely populated region of Victoria has increased by 75% between 2000 and 2010.^21^

The Nordic countries have witnessed an increase in the number of law-abiding youths from 1994 and 2008.^22^ In Sweden, both objective levels of juvenile crime^23^ and self-reported involvement in juvenile crime^24^ have fallen between 1995 and 2005. Similarly in Finland, where, despite fluctuating trends in juvenile drug use, juvenile property and violent crime is reported to have decreased between 1992 and 2013.^25^

To summarise, whereas regional and annual trends in juvenile offending are observed and expected, a global trend characterised by decreased juvenile offending appears to have emerged in recent years. Indeed, UN data from a sample of 40 countries lend support to this conclusion, indicating a decrease in the proportion of juveniles suspected (10.9% to 9.2%) and convicted (7.5% to 6%) of crime between 2004 and 2012, respectively.^26^

### Juvenile gang membership

#### Influence on crime involvement

One of the features of urbanisation across the world has been the rise of youth gangs, groups of young people often defined by geographical area, ethnic identity or ideology; recent reports indicate a rise in groups with extremist views. Explanatory models for the rise in youth gangs include factors such as economic migration, loss of extended family networks, reduced supervision of children, globalisation and exposure to inaccessible lifestyle ‘ideals’ portrayed in modern media.

Authorities in Japan attributed a surge in serious youth crime in the 1990s primarily to juvenile bike gangs known as ‘bosozoku’, who were deemed responsible for over 80% of serious offences perpetrated by juveniles, putatively bolstered by a crackdown on yakuza organised crime syndicates.^27^ Although difficult to quantify, gang involvement appears to feature in a large proportion of juvenile offences, and there is evidence that gang membership has a facilitating effect on perpetration of the most serious violence including homicide.^28^

#### Mental health

Compared with general and juvenile offender populations, juvenile gang members exhibit significantly higher rates of mental health problems such as conduct disorder/antisocial personality disorder, post-traumatic stress disorder (PTSD), anxiety disorders and attention-deficit hyperactivity disorder (ADHD).^29^ Gang members, compared with non-violent men who do not belong to a gang, are far more likely to utilise mental health services and display significantly higher levels of psychiatric morbidity, most notably antisocial personality disorder, psychosis and anxiety disorders.^30^ Gang membership has also been positively correlated with an increased incidence of depressed mood and suicidal ideation among younger gang members.^31^ Prevalence of ADHD is significantly greater in incarcerated youth populations (30.1%) than in general youth population estimates (3–7%),^32^ therefore it may be reasonable to expect a similarly increased prevalence in juvenile gang members. ADHD has also been associated with a significantly increased risk of comorbid mood/affective disorder.^33^

## Forensic child and adolescent psychiatric services

Increased awareness of constitutional and environmental factors that contribute to juvenile offending has strengthened a public health perspective towards the problem, and in the UK entry into the youth justice system has been adopted as an indicator of general public health.^34^

Dictionaries frequently define ‘forensic’ as meaning ‘legal’, implying a relationship with any court of law. Indeed, many forensic psychiatrists, particularly in child and adolescent services, undertake roles that encompass multiple legal domains relevant to mental health, including criminal law, family and child custody proceedings, special educational tribunals, and immigration or extradition matters.

Specialist forensic psychiatric services vary considerably between countries,^35^ but usually forensic psychiatrists assess and treat individuals in secure psychiatric hospitals, prisons, law courts, police stations and in the community under various levels of security, supervision and support. In some countries there has been a trend towards forensic psychiatrists working almost exclusively with courts of law, providing independent specialist opinion to assist the court.

In the UK, forensic child and adolescent psychiatry has emerged as a clinical subspecialty. Some services are based in specialist secure hospitals for young people and cater for the relatively small number of high-risk young offenders with the most severe mental disorders. In the absence of such specialist resources, young people may be managed in suboptimal environments such as juvenile prisons, secure residential placements or secure mental health wards for adults, or even fail to receive treatment at all.

In light of growing evidence-based interventions for juvenile offenders within a public health framework,^36^ the role of child and family mental health services may increase over time. Aside from direct clinical roles, practitioners in forensic child and adolescent psychiatry are also well placed to work with a wide range of partner agencies on the planning and delivery of broader interventions for the primary and secondary prevention of juvenile delinquency.

## Treatment

### Prevalence of mental health problems among juvenile offenders

Rates of mental health problems among juvenile offenders are significantly higher than in their non-offender peers, with two-thirds of male juvenile offenders in the USA suggested as meeting criteria for at least one psychiatric disorder.^37^ One in five juvenile offenders is estimated to suffer severe functional impairment as a result of their mental health problems.^38^ Paradoxically, these needs are often unmet,^39,40^ despite evidence of increased contact with mental health services, particularly among first-time juvenile offenders.^41,42^ Of additional concern are the reported associations between mental health problems and mortality in incarcerated juveniles,^43^ including an elevated suicide rate for males.^44^ Mental health problems must be a target in interventions for juvenile offenders; however, treatments which focus solely on clinical problems are unlikely to result in benefit for criminogenic outcomes.^45^ There is therefore a clear need for effective interventions which address both the clinical and criminogenic needs of these individuals.

### Evidence-based treatments for mental health problems

#### Treatment of PTSD

Estimates regarding the prevalence of PTSD among juvenile offenders suggest that 20 to 23% meet the clinical criteria,^46,47^ with prevalence rates significantly higher among females than males (40% *v*. 17%).^46^ Moreover, with 62% experiencing trauma within the first 5 years of life^47^ and up to 93% experiencing at least one traumatic event during childhood or adolescence,^48^ this should be a target for intervention.

Cognitive–behavioural therapy (CBT) is regarded as the most effective intervention for adults with PTSD^49^ and also has demonstrated efficacy for juvenile non-offenders.^50,51^ There is limited evidence suggesting a significant reduction in self-reported symptoms of PTSD following group-based CBT in male juvenile offenders,^52^ and of an adapted version of CBT, cognitive processing therapy,^53^ also resulting in a significant reduction in self-reported symptoms of PTSD and depression compared with waitlist controls.^54^

A trauma-focused emotion regulation intervention (TARGET) has received preliminary empirical support for use in this population. TARGET resulted in nearly twice as much reduction in PTSD symptom severity as treatment as usual (TAU),^55^ in addition to significant reductions in depression, behavioural disturbances and increased optimism.^56^

#### Mood/anxiety disorders and self-harm

Juvenile offenders in the UK present with a high prevalence of mood and anxiety disorders (67% of females, 41% of males), self-harm (11% of females, 7% of males) and history of suicide attempts (33% of females, 20% of males).^57^ Similarly high prevalence has also been observed cross-culturally, namely in the USA,^37,58^ Switzerland^59^ and Finland.^60^

Despite such high prevalence, there appears to be a paucity of high-quality evaluations regarding the effectiveness of interventions for juvenile offenders with mood and/or anxiety disorders, or problems with self-harm. However, the limited evidence that is available suggests that group-based CBT may aid symptom reduction.^61^ Recovery rates for major depressive disorder following group-based CBT are over double those for a life skills tutoring intervention (39% *v*. 19%, respectively), although no significant difference was noted at 6- or 12-month follow-up. CBT also resulted in significantly greater improvements in self- and observer-reported symptoms of depression and social functioning.^62^

However, group-based CBT is not reported to be significantly different from TAU in reduction of self-harm,^63^ whereas individual CBT is not significantly different from TAU in outcomes for depression, anxiety, conduct disorder or PTSD.^64^ Yet recruitment to and retention in intervention seems good, suggesting that CBT is feasible to implement in juvenile offender populations.^64^

Evaluations of alternative interventions have posited muscle relaxation as effective in improving juvenile offenders' tolerance of frustration.^65^ Dialectical behaviour therapy (DBT) has also been reported to significantly reduce incidences of physical aggression in a juvenile offender population^66^ and among juvenile non-offenders expressing suicidal ideation.^67^ It significantly reduced serious behavioural problems and staff punitive actions among juvenile offenders within a mental health unit, although no similar significant reductions were observed for those without mental health problems.^68^

#### Evidence-based treatments for conduct disorder: family approaches

Relationships with family and peers are recognised as key factors in the criminogenic profile of juvenile offenders.^69^ Multisystemic therapy (MST) is a family-focused intervention targeting characteristics related to antisocial behaviour, including family relationships and peer associations,^70^ with evidence from US and UK studies suggesting MST is a beneficial intervention for juvenile offenders. When compared with conventional services offered by juvenile offending services, MST was associated with a significant reduction in the likelihood of reoffending,^71^ maintained 2 and 4 years post-treatment.^72,73^ Offenders engaging in MST are reported to be significantly less likely to become involved in serious and violent offending.^73,74^ Significant improvements have also been observed in both self- and parent-reported delinquency,^74^ family relations and interactions,^73^ and home, school, community and emotional functioning.^71^ A cost offset analysis of MST among UK juvenile offenders suggested that combining MST and conventional services provides greater cost savings than conventional services alone, as a result of its positive effects on recidivism.^75^ Qualitative impressions of MST from juvenile offenders and their parents indicate that key components of a successful delivery of MST include the quality of the therapeutic relationship and ability to re-engage the offender with educational systems.^76^

Some evidence also exists regarding the efficacy of MST when delivered to non-offender antisocial juvenile populations outside the USA and the UK. Compared with TAU, MST resulted in a significantly greater increase in social competence and caregiver satisfaction, and a significant reduction in referrals for out-of-home placements, in Norwegian juveniles exhibiting serious behavioural problems.^77^ However, no significant difference between MST and TAU was reported in outcomes for antisocial behaviour and psychiatric symptoms in Swedish juvenile offenders.^78^ MST was also found to have no significant benefit over TAU in outcomes including recidivism in a sample of Canadian juvenile offenders.^79^ These differing outcomes have been posited as the result of barriers in transferring MST from US and UK populations owing to differing approaches to juvenile justice between countries (i.e. a welfare *v*. justice approach).^78^ The heterogeneous nature of studies concerning MST in juvenile offender populations prevent a firm conclusion being drawn as to its superiority over alternative interventions, although this does not diminish the positive outcomes which have been observed.^80^

### Substance misuse

Motivational interviewing represents a promising approach for juvenile offenders, particularly as a treatment for substance misuse.^81^ Group-based motivational interviewing has received positive feedback from participants when implemented with first-time juvenile alcohol or drug offenders,^82^ and compared with TAU, juvenile offenders in receipt of motivational interviewing have greater satisfaction and display lower, though not statistically significant, rates of recidivism at 12-months post-motivational interviewing.^83^ There is therefore preliminary evidence for the acceptability and feasibility of motivational interviewing for substance-misusing juvenile offenders, but future research regarding long-term outcomes is warranted. To date, motivational interviewing for difficulties faced by juvenile offenders beyond that of substance misuse does not appear to have received much research attention. Juvenile offenders are known for their difficulty to engage in rehabilitative services, therefore further investigation of the effectiveness of motivational interviewing in encouraging engagement is warranted.

Preliminary investigations have also developed a conceptual framework for the delivery of mindfulness-based interventions (MBI) to incarcerated substance-misusing juveniles, with qualitative impressions suggesting this is a potentially feasible and efficacious intervention.^84^ Although literature regarding the effectiveness of MBI in juvenile offenders is scarce, qualitative feedback has indicated positive reception of this style of intervention, with particular improvements in subjective well-being reported by juvenile participants.^85^

### Employment and education

Engaging juvenile offenders with education and skills-based training is an important component of successful rehabilitation, with positive engagement in meaningful activities associated with improvements in areas such as self-belief^86^ and protection against future participation in criminal activities.^87^ It is concerning therefore that an evaluation of the use of leisure time over a 1-week period by probationary juvenile offenders in Australia indicated only 10% of this time was spent engaging in productive activities, such as employment or education, with 57% used for passive leisure activities, a level 30% higher than that of their non-offender peers.^88^

Efforts to engage juvenile offenders in vocational and/or occupational activities have shown benefits in a number of areas. A specialised vocational and employment training programme (CRAFT) emphasising practical skills was evaluated against conventional education provision to juvenile offenders in the USA. Over a 30-month follow-up period, those engaged in CRAFT were significantly more likely to be in employment, to have attended an educational diploma programme and to have attended for a significantly longer period of time.^89^ Benefits have also been reported with regard to risk of reoffending, with an after-school programme in the USA incorporating practical community projects, educational sessions and family therapy resulting in a significant reduction in recidivism at 1-year follow-up.^90^

Qualitative investigations of US juvenile offenders suggest there is not a lack of interest in pursuing education among this population, but rather a disconnection with educational systems when education providers are perceived not to care about students' progress.^91^ Ensuring education providers are perceived as proactive and caring in this regard may therefore be an important consideration for efforts to engage juvenile offenders with educational systems. Significant barriers to engagement include difficulties in obtaining accurate information regarding the offender's educational history, in addition to identifying community-based education providers willing to accept previously incarcerated juveniles on their release.^92^

### Language and communication

Difficulties with language and communication skills appear to be prevalent among juvenile offenders, with estimates of those falling into the poor or very poor categories ranging from 46 to 67%; overall, up to 90% of juvenile offenders demonstrated language skills below average.^93^ Specifically, high rates of illiteracy are reported in this population,^94^ with evidence to suggest that an awareness of such problems among juvenile offenders themselves is associated with dissatisfaction and poor self-esteem.^95^ These difficulties may act as barriers to engagement in therapeutic interventions, particularly those delivered in group settings, as well as re-engagement with educational systems. Awareness of the challenges these young people face with regard to confidence and ability to communicate is important, and potential involvement of a speech and language therapist could be considered. Preventing deficits in language and communication through effective schooling and appropriate support in the early years of life may serve as an aid to effective engagement in rehabilitative interventions, and may also mitigate the risk of engagement in criminal activities in the first instance.

## Delivery of therapeutic services

### Common challenges to a therapeutic youth justice pathway

There are common obstacles to smooth care pathways between different parts of systems, such as in transitions between secure settings and the community, between prisons and secure psychiatric settings, and between child and adult services. In some jurisdictions individuals can only be treated pharmacologically against their will in a hospital setting, a safeguard which limits the extent to which individuals can be treated in prison, but there is still great scope for intervention by prison mental health teams in juvenile prisons.

### Factors associated with good outcomes

A meta-analysis has revealed three primary factors associated with effective interventions for juvenile offenders: a ‘therapeutic’ intervention philosophy, serving high-risk offenders, and quality of implementation.^96^ These findings are consistent with factors posited as correlating with good outcome in residential centres for troubled adolescents and juvenile offenders: good staff-adolescent relations, perception of staff as pro-social role models, positive peer pressure, an individualised therapeutic programme approach, developmentally appropriate programmes and activities, clear expectations and boundaries, and placement locations which allow for continued family contact.^97,98^

In the community, coercive styles of engagement have been found to be less successful at achieving adherence among juvenile offenders than a client-centred approach.^99^

### Factors associated with poor outcomes

‘Scared Straight’ programmes expose juveniles who have begun to commit offences to inmates of high-security prisons, yet these approaches have been discredited due to evidence that risk of recidivism may in fact increase following such exposure.^100^ Similarly poor outcomes have been observed in programmes modelled on military boot camps, in which harsh discipline is considered to be of therapeutic benefit,^101^ and initiatives such as curfew, probation and hearing juvenile cases in adult court were also shown to be ineffective in reducing recidivism.^13^

Over recent years it has been repeatedly demonstrated that exposure to juvenile court itself appears to have a detrimental effect on juvenile offending.^102–104^ This may be partially explained by effects of labelling, stigma and negative self-image associated with a criminal conviction, but also the practical consequences of sentences, including assortment of delinquent peers in community or prison sentences. Incarceration presents several additional harms, including disturbance of care and pro-social relationships, discontinuity in education, association with delinquent peers, and exposure to violence. Half of detained young offenders in the UK reported victimisation during their current prison term,^57^ while 12% of incarcerated youth in the USA reported sexual victimisation in the previous year.^105^ International agreements state that deprivation of liberty (such as juvenile prison) should be used as a last resort and for the shortest time necessary, so should be reserved for the highest-risk offenders. The cost of juvenile antisocial behaviour is known to be high, and to fall on many agencies.^106^ The current climate of austerity in public services demands that any interventions should be not only effective, but also cost-effective, raising a clear challenge – and opportunity – for the implementation of interventions for this population of vulnerable young people. For example, parenting programmes have demonstrated sustained benefits for this population,^107,108^ with economic analysis indicating gross savings of £9288 per child over a 25 year period.^109^ Considered together with wider costs of crime, these gross savings exceed the average cost of parenting programmes (£1177) by a factor of approximately 8 to 1.

## Conclusions

Many argue that we have a long way to go before arriving at ‘child friendly’ juvenile justice.^110^ Around the world there are variable and inadequate legal frameworks that are not age-appropriate, there is a lack of age-appropriate services and establishments, and a lack of a specialist workforce, leading to challenges around training and supervision to work with this vulnerable population. In the UK and other high-income countries worldwide, forensic child and adolescent psychiatry is a multifaceted discipline incorporating legal, psychiatric and developmental fields. This approach has navigated clinical and ethical challenges and made an important contribution to welfare and justice needs by its adoption of an evidence-based therapeutic intervention philosophy.

## References

[1] Muncie J Goldson B Comparative Youth Justice. Sage, 2006.

[2] Fougere A Thomas S Daffern M A study of the multiple and complex needs of Australian young adult offenders. Austral Psychologist 2013; 48: 188–95.

[3] Hughes N Williams H Chitsabesan P Davies R Mounce L Nobody Made the Connection: The Prevalence of Neurodisability in Young People who Offend. Office of the Children's Commissioner, 2012.

[4] Lyons JS Royce Baerger D Quigley P Erlich J Griffin E Mental health service needs of juvenile offenders: a comparison of detention, incarceration, and treatment settings. Child Serv Soc Policy Res Pract 2001; 4: 69–85.

[5] Kinner SA Degenhardt L Coffey C Sawyer S Hearps S Patton G Complex health needs in the youth justice system: a survey of community-based and custodial offenders. J Adolesc Health 2014; 54: 521–6. 24287014 10.1016/j.jadohealth.2013.10.003

[6] Robertson AA Dill PL Husain J Undesser C Prevalence of mental illness and substance abuse disorders among incarcerated juvenile offenders in Mississippi. Child Psychiatry Hum Dev 2004; 35: 55–74. 15626325 10.1023/b:chud.0000039320.40382.91

[7] Penner EK Roesch R Viljoen JL Young offenders in custody: an international comparison of mental health services. Int J Forens Ment Health 2011; 10: 215–32.

[8] Penal Reform International, UK Aid The Minimum Age of Criminal Responsibility (Justice for Children Briefing No. 4). Penal Reform International, 2013.

[9] Forsyth CJ Asmus G Forsyth YA Stokes BR Mayne M Child delinquents: examining the market for criminal futures. Dev Behav 2011; 32: 441–50.

[10] World Health Organization The ICD-10 Classification of Mental and Behavioural Disorders: Clinical Descriptions and Diagnostic Guidelines. WHO, 1992.

[11] Piquero A Steinberg L Rehabilitation Versus Incarceration of Juvenile Offenders: Public Preferences in Four Models for Change States. MacArthur Research Network on Adolescent Development and Juvenile Justice, 2008.

[12] Hazel AN Cross-National Comparison of Youth Justice. Youth Justice Board, 2008.

[13] Stevens A Kessler I Gladstone B Review of Good Practices in Preventing Juvenile Crime in the European Union. University of Kent & European Crime Prevention Network, 2006 Available at http://www.eucpn.org/library/index.asp (accessed 13 June 2013).

[14] Streib VL The Juvenile Death Penalty Today: Death Sentences and Executions for Juvenile Crimes, January 1, 1973-December 31, 2004. National Criminal Justice Reference Service, 2005.

[15] *Roper v Simmons*, 543 U.S. 112 (2005).

[16] Satcher D Youth *Violence: A Report of the Surgeon General*. Department of Health and Human Services, 2001.

[17] Browne A Williams KR Parker RN Strom KJ Barrick K Youth homicide in the United States. In Encyclopedia of Criminology and Criminal Justice: 5585–95. Springer New York, 2014.

[18] Ministry of Justice Youth Justice Board for England and Wales Youth Justice Statistics 2014/15, England and Wales. Youth Justice Board/Ministry of Justice Statistics Bulletin. Youth Justice Board, 2016 Available at https://www.gov.uk/government/statistics/youth-justice-annual-statistics-2014-to-2015 (accessed 12 February 2016).

[19] Bateman T Where has all the youth crime gone? Youth justice in an age of austerity. Child Soc 2014; 28: 416–24.

[20] Australian Bureau of Statistics Recorded Crime – Offenders, Australia 2013–14 (cat. no. 4519.0). Australian Bureau of Statistics, 2016 Available at http://www.abs.gov.au/ausstats/abs@.nsf/mf/4519.0 (accessed 5 August 2015).

[21] Papalia N Thomas SD Ching H Daffern M Changes in the prevalence and nature of violent crime by youth in Victoria, Australia. Psychiatr Psychol Law 2015; 22: 213–23.

[22] Kivivuori J Bernburg JG Delinquency research in the Nordic countries. Crime Justice 2011; 40: 405–77.

[23] Shannon D Bäckman O Estrada F Nilsson A Youth and Crime in Sweden. Oxford University Press, 2014.

[24] Svensson R Ring J Trends in self-reported youth crime and victimization in Sweden, 1995–2005. J Scand Stud Crim Crime Prev 2007; 8: 185–209.

[25] Elonheimo H Evidence for the crime drop: survey findings from two Finnish cities between 1992 and 2013. J Scand Stud Crim Crime Prev 2014; 15: 209–17.

[26] United Nations Economic and Social Council World crime Trends and Emerging Issues and Responses in the Field of Crime Prevention and Criminal Justice. Commission on Crime Prevention and Criminal Justice, 2014 (https://www.unodc.org/documents/data-and-analysis/statistics/crime/ECN.1520145_EN.pdf).

[27] Kattoulas V Young, fast and deadly. Far East Econ Rev 2001; 164: 64–7.

[28] Rosenfeld R Bray TM Egley A Facilitating violence: a comparison of gang-motivated, gang-affiliated, and non-gang youth homicides. J Quant Crim 1999; 15: 495–516.

[29] Madden V Understanding the Mental Health Needs of Young People Involved in Gangs: A Tri-Borough Public Health Report Produced on Behalf of the Westminster Joint Health and Wellbeing Board. The Westminster Joint Health and Wellbeing Board, 2013.

[30] Coid JW Ullrich S Keers R Bebbington P DeStavola BL Kallis C Gang membership, violence, and psychiatric morbidity. Am J Psychiatry 2013; 170: 985–93. 23846827 10.1176/appi.ajp.2013.12091188

[31] McDaniel DD Risk and protective factors associated with gang affiliation among high-risk youth: a public health approach. Injury Prev 2012; 18: 253–8. 10.1136/injuryprev-2011-040083 PMC3406611 22240265

[32] Young S Moss D Sedgwick O Fridman M Hodgkins P A meta-analysis of the prevalence of attention deficit hyperactivity disorder in incarcerated populations. Psychol Med 2015; 45: 247–58. 25066071 10.1017/S0033291714000762 PMC4301200

[33] Young S Sedgwick O Fridman M Gudjonsson G Hodgkins P Lantigua M Co-morbid psychiatric disorders among incarcerated ADHD populations: a meta-analysis. Psychol Med 2015; 45: 2499–510. 25857258 10.1017/S0033291715000598 PMC4531473

[34] Department of Health A Public Health Framework for England, 2013–2016: Part 1. Department of Health, 2012.

[35] Gordon H Lindqvist P Forensic psychiatry in Europe. Psychiatr Bull 2007; 31: 421–4.

[36] Cocozza JJ Skowyra KR Youth with mental health disorders: issues and emerging responses. Juvenile Justice 2000; 7: 3–13.

[37] Teplin LA Abram KM McClelland GM Dulcan MK Mericle AA Psychiatric disorders in youth in juvenile detention. Arch Gen Psychiatry 2002; 59: 1133–43. 12470130 10.1001/archpsyc.59.12.1133 PMC2861992

[38] Hammond S Mental Health Needs of Juvenile Offenders. National Conference of State Legislatures, 2007.

[39] Lennox C The health needs of young people in prison. Br Med Bull 2014; 112: 17–25. 25267312 10.1093/bmb/ldu028

[40] McGorry P Bates T Birchwood M Designing youth mental health services for the 21st century: examples from Australia, Ireland and the UK. Br J Psychiatry 2013; 202: 30–5. 10.1192/bjp.bp.112.119214 23288499

[41] Burke JD Mulvey EP Schubert CA Prevalence of mental health problems and service use among first-time juvenile offenders. J Child Fam Stud 2015; 24: 3774–81. 26557012 10.1007/s10826-015-0185-8 PMC4635474

[42] Kataoka SH Zima BT Dupre DA Moreno KA Yang X McCracken JT Mental health problems and service use among female juvenile offenders: their relationship to criminal history. J Am Acad Child Adolesc Psychiatry 2001; 40: 549–55. 11349699 10.1097/00004583-200105000-00014

[43] Sailas ES Feodoroff B Lindberg NC Virkkunen ME Sund R Wahlbeck K The mortality of young offenders sentenced to prison and its association with psychiatric disorders: a register study. Eur J Public Health 2006; 16: 193–7. 16141298 10.1093/eurpub/cki169

[44] Fazel S Benning R Danesh J Suicides in male prisoners in England and Wales, 1978–2003. Lancet 2005; 366: 1301–2. 16214601 10.1016/S0140-6736(05)67325-4

[45] Schubert CA Mulvey EP Glasheen C Influence of mental health and substance use problems and criminogenic risk on outcomes in serious juvenile offenders. J Am Acad Child Adolesc Psychiatry 2011; 50: 925–37. 21871374 10.1016/j.jaac.2011.06.006

[46] Moore E Gaskin C Indig D Childhood maltreatment and post-traumatic stress disorder among incarcerated young offenders. Child Abuse Negl 2013; 37: 861–70. 23978574 10.1016/j.chiabu.2013.07.012

[47] Dierkhising CB Ko SJ Woods-Jaeger B Briggs EC Lee R Pynoos RS Trauma histories among justice-involved youth: findings from the National Child Traumatic Stress Network. Eur J Psychotraumatol 2013; doi: 10.3402/ejpt.v4i0.20274. 10.3402/ejpt.v4i0.20274 PMC3714673 23869252

[48] Wilson HW Berent E Donenberg GR Emerson EM Rodriguez EM Sandesara A Trauma history and PTSD symptoms in juvenile offenders on probation. Vict Offender 2013; 8: 465–77. 10.1080/15564886.2013.835296 PMC3834597 24273468

[49] Gallagher MW Thompson-Hollands J Bourgeois ML Bentley KH Cognitive behavioral treatments for adult posttraumatic stress disorder: current status and future directions. J Contemp Psychother 2015; 45: 235–43.

[50] Deblinger E Mannarino AP Cohen JA Steer RA A follow-up study of a multisite, randomized, controlled trial for children with sexual abuse-related PTSD symptoms. J Am Acad Child Adolesc Psychiatry 2006; 45: 1474–84. 17135993 10.1097/01.chi.0000240839.56114.bb

[51] Nixon RDV Sterk J Pearce A A randomized trial of cognitive behaviour therapy and cognitive therapy for children with posttraumatic stress disorder following single-incident trauma. J Abnorm Child Psychol 2012; 40: 327–37. 21892594 10.1007/s10802-011-9566-7

[52] Ovaert LB Cashel ML Sewell KW Structured group therapy for posttraumatic stress disorder in incarcerated male juveniles. Am J Orthopsychiatry 2003; 73: 294–301. 12921210 10.1037/0002-9432.73.3.294

[53] Resick PA Schnicke MK Cognitive processing therapy for sexual assault victims. J Consult Clin Psychol 1992; 60: 748. 1401390 10.1037//0022-006x.60.5.748

[54] Ahrens J Rexford L Cognitive processing therapy for incarcerated adolescents with PTSD. J Aggression Maltreat Trauma 2002; 6: 201–16.

[55] Ford JD Steinberg KL Hawke J Levine J Zhang W Randomized trial comparison of emotion regulation and relational psychotherapies for PTSD with girls involved in delinquency. J Clin Child Adolesc Psychol 2012; 41: 27–37. 22233243 10.1080/15374416.2012.632343

[56] Marrow MT Knudsen KJ Olafson E Bucher SE The value of implementing TARGET within a trauma-informed juvenile justice setting. J Child Adolesc Trauma 2012; 5: 257–70.

[57] Lader D Singleton N Meltzer H Psychiatric Morbidity Among Young Offenders in England and Wales. Office for National Statistics, 2000. 10.1080/0954026021000046074 12745323

[58] Shufelt JL Cocozza JJ Youth with Mental Health Disorders in the Juvenile Justice System: Results from a Multi-State Prevalence Study. National Center for Mental Health and Juvenile Justice, 2006.

[59] Gisin D Haller DM Cerutti B Wolff H Bertrand D Sebo P Mental health of young offenders in Switzerland: recognizing psychiatric symptoms during detention. J Forens Legal Med 2012; 19: 332–6. 10.1016/j.jflm.2012.02.013 22847050

[60] Sailas ES Feodoroff B Virkkunen M Wahlbeck K Mental disorders in prison populations aged 15-21: national register study of two cohorts in Finland. BMJ 2005; 330: 1364–5. 15829486 10.1136/bmj.38415.633762.F7 PMC558286

[61] Townsend E Walker DM Sargeant S Vostanis P Hawton K Stocker O Systematic review and meta-analysis of interventions relevant for young offenders with mood disorders, anxiety disorders, or self-harm. J Adolesc 2010; 33: 9–20. 19560808 10.1016/j.adolescence.2009.05.015

[62] Rohde P Clarke GN Mace DE Jorgensen JS Seeley JR An efficacy/effectiveness study of cognitive-behavioral treatment for adolescents with comorbid major depression and conduct disorder. J Am Acad Child Adolesc Psychiatry 2004; 43: 660–8. 15167082 10.1097/01.chi.0000121067.29744.41

[63] Rohde P Jorgensen JS Seeley JR Mace DE Pilot evaluation of the coping course: a cognitive-behavioral intervention to enhance coping skills in incarcerated youth. J Am Acad Child Adolesc Psychiatry 2004; 43: 669–76. 15167083 10.1097/01.chi.0000121068.29744.a5

[64] Mitchell P Smedley K Kenning C McKee A Woods D Rennie CE Cognitive behaviour therapy for adolescent offenders with mental health problems in custody. J Adolesc 2011; 34: 433–43. 20673994 10.1016/j.adolescence.2010.06.009

[65] Nakaya N Kumano H Minoda K Koguchi T Tanouchi K Kanazawa M Preliminary study: psychological effects of muscle relaxation on juvenile delinquents. Int J Behav Med 2004; 11: 176–80. 15496345 10.1207/s15327558ijbm1103_6

[66] Shelton D Kesten K Zhang W Trestman R Impact of a dialectic behavior therapy-corrections modified (DBT-CM) upon behaviorally challenged incarcerated male adolescents. J Child Adolesc Psychiatr Nurs 2011; 24: 105–13. 21501287 10.1111/j.1744-6171.2011.00275.x PMC3080237

[67] Katz LY Cox BJ Gunasekara S Miller AL Feasibility of dialectical behavior therapy for suicidal adolescent inpatients. J Am Acad Child Adolesc Psychiatry 2004; 43: 276–82. 15076260 10.1097/00004583-200403000-00008

[68] Trupin EW Stewart DG Beach B Boesky L Effectiveness of a dialectical behaviour therapy program for incarcerated female juvenile offenders. Child Adolesc Ment Health 2002; 7: 121–7.

[69] Andrews DA Bonta J The Psychology of Criminal Conduct. Routledge, 2010.

[70] Henggeler SW Melton GB Smith LA Family preservation using multisystemic therapy: an effective alternative to incarcerating serious juvenile offenders. J Consult Clin Psychol 1992; 60: 953–61. 1460157 10.1037//0022-006x.60.6.953

[71] Timmons-Mitchell J Bender MB Kishna MA Mitchell CC An independent effectiveness trial of multisystemic therapy with juvenile justice youth. J Clin Child Adolesc Psychol 2006; 35: 227–36. 16597218 10.1207/s15374424jccp3502_6

[72] Henggeler SW Melton GB Smith LA Schoenwald SK Hanley JH Family preservation using multisystemic treatment: long-term follow-up to a clinical trial with serious juvenile offenders. J Child Fam Stud 1993; 2: 283–93.

[73] Borduin CM Mann BJ Cone LT Henggeler SW Fucci BR Blaske DM Multisystemic treatment of serious juvenile offenders: long-term prevention of criminality and violence. J Consult Clin Psychol 1995; 63: 569. 7673534 10.1037//0022-006x.63.4.569

[74] Butler S Baruch G Hickey N Fonagy P A randomized controlled trial of multisystemic therapy and a statutory therapeutic intervention for young offenders. J Am Acad Child Adolesc Psychiatry 2011; 50: 1220–35. 22115143 10.1016/j.jaac.2011.09.017

[75] Cary M Butler S Baruch G Hickey N Byford S Economic evaluation of multisystemic therapy for young people at risk for continuing criminal activity in the UK. PloS ONE 2013; 8: e61070. 23613786 10.1371/journal.pone.0061070 PMC3632567

[76] Tighe A Pistrang N Casdagli L Baruch G Butler S Multisystemic therapy for young offenders: families' experiences of therapeutic processes and outcomes. J Fam Psychol 2012; 26: 187. 22329390 10.1037/a0027120

[77] Ogden T Halliday-Boykins CA Multisystemic treatment of antisocial adolescents in Norway: replication of clinical outcomes outside of the US. Child Adolesc Ment Health 2004; 9: 77–83. 10.1111/j.1475-3588.2004.00085.x 32797502

[78] Sundell K Hansson K Löfholm CA Olsson T Gustle LH Kadesjö C The transportability of multisystemic therapy to Sweden: short-term results from a randomized trial of conduct-disordered youths. J Fam Psychol 2008; 22: 550. 18729669 10.1037/a0012790

[79] Cunningham A One Step Forward: Lesson Learned from a Randomized Study of Multisystemic Therapy in Canada. Centre for Children and Families in the Justice System, 2002.

[80] Littell JH Campbell M Green S Toews B Multisystemic therapy for social, emotional, and behavioral problems in youth aged 10–17. Cochrane Database Syst Rev 2005; 4: CD004797. 10.1002/14651858.CD004797.pub4 PMC13591134 16235382

[81] Feldstein SW Ginsburg JI Motivational interviewing with dually diagnosed adolescents in juvenile justice settings. Brief Treat Crisis Interventions 2006; 6: 218.

[82] D'Amico EJ Osilla KC Hunter SB Developing a group motivational interviewing intervention for first-time adolescent offenders at-risk for an alcohol or drug use disorder. Alcohol Treat Quart 2010; 28: 417–36. 10.1080/07347324.2010.511076 PMC2991057 21113392

[83] D'Amico EJ Hunter SB Miles JN Ewing BA Osilla KC A randomized controlled trial of a group motivational interviewing intervention for adolescents with a first time alcohol or drug offense. J Subst Abuse Treat 2013; 45: 400–8. 23891459 10.1016/j.jsat.2013.06.005 PMC3826597

[84] Himelstein S Saul S Garcia-Romeu A Pinedo D Mindfulness training as an intervention for substance user incarcerated adolescents: a pilot grounded theory study. Subst Use Misuse 2014; 49: 560–70. 24611851 10.3109/10826084.2013.852580

[85] Himelstein S Hastings A Shapiro S Heery M A qualitative investigation of the experience of a mindfulness-based intervention with incarcerated adolescents. Child Adolesc Ment Health 2012; 17: 231–7. 10.1111/j.1475-3588.2011.00647.x 32847276

[86] DeForest D Watts JH Madigan MJ Resonation in the model of human occupation: a pilot study. Occup Ther Ment Health 1991; 11: 57–71.

[87] Hoge RD Andrews DA Leschied AW An investigation of risk and protective factors in a sample of youthful offenders. J Child Psychol Psychiatry 1996; 37: 419–24. 8735441 10.1111/j.1469-7610.1996.tb01422.x

[88] Farnworth L Time use and leisure occupations of young offenders. Am J Occup Ther 2000; 54: 315–25. 10842688 10.5014/ajot.54.3.315

[89] Schaeffer CM Henggeler SW Ford JD Mann M Chang R Chapman JE RCT of a promising vocational/employment program for high-risk juvenile offenders. J Subst Abuse Treat 2014; 46: 134–43. 23958035 10.1016/j.jsat.2013.06.012 PMC3840119

[90] Myers WC Burton PR Sanders PD Donat KM Cheney J Fitzpatrick TM Project back-on-track at 1 year: a delinquency treatment program for early-career juvenile offenders. J Am Acad Child Adolesc Psychiatry 2000; 39: 1127–34. 10986809 10.1097/00004583-200009000-00012

[91] Reed DK Wexler J ‘Our teachers … don't give us no help, no nothin”: juvenile offenders' perceptions of academic support. Resident Treat Child Youth 2014; 31: 188–218.

[92] Smeets E Education in young offender institutions and secure youth care institutions Educ Res Eval 2014; 20: 67–80.

[93] Bryan K Freer J Furlong C Language and communication difficulties in juvenile offenders. Int J Lang Commun Disord 2007; 42: 505–20. 17729143 10.1080/13682820601053977

[94] Hodges J Giuliotti N Porpotage FM II Improving Literacy Skills of Juvenile Detainees. Juvenile Justice Bulletin, Office of Juvenile Justice and Delinquency Prevention, 1994.

[95] Hopkins T Clegg J Stackhouse J Young offenders' perspectives on their literacy and communication skills. Int J Lang Commun Disord 2016; 51: 95–109. 26344238 10.1111/1460-6984.12188

[96] Lipsey MW The primary factors that characterize effective interventions with juvenile offenders: a meta-analytic overview. Vict Offend 2009; 4: 124–47.

[97] Sedlak A McPherson KS Conditions of Confinement: Findings from the Survey of Youth in Residential Placement. US Department of Justice, Office of Justice Programs, Office of Juvenile Justice and Delinquency Prevention, 2010.

[98] Pazaratz D Skills training for managing disturbed adolescents in a residential treatment program. Clin Child Psychol Psychiatry 2003; 8: 119–30.

[99] Schwalbe CS Maschi T Confronting delinquency: probations officers' use of coercion and client-centered tactics to foster youth compliance. Crim Delinq 2011; 57: 801–22.

[100] Petrosino A Turpin-Petrosino C Hollis-Peel ME Lavenberg JG ‘Scared Straight’ and other juvenile awareness programs for preventing juvenile delinquency. Cochrane Database Syst Rev 2013; 4: CD002796. 10.1002/14651858.CD002796.pub2 PMC11973542 23862186

[101] Wilson DB MacKenzie DL Mitchell FN Effects of Correctional Boot Camps on Offending (Campbell Systematic Review). The Campbell Collaboration, 2008.

[102] McAra L McVie S Youth justice? The impact of system contact on patterns of desistance from offending. Eur J Crim 2007; 4: 315–45.

[103] Petrosino A Turpin-Petrosino C Guckenburg S Formal system processing of juveniles: effects on delinquency. Campbell Syst Rev 2010; doi:10.4073/csr.20101.

[104] Petitclerc A Gatti U Vitaro F Tremblay RE Effects of juvenile court exposure on crime in young adulthood. J Child Psychol Psychiatry 2013; 54: 291–7. 23009564 10.1111/j.1469-7610.2012.02616.x

[105] Beck AJ Harrison PM Guerino P *Sexual Victimization in Juvenile Facilities Reported by Youth, 2008–09* (NCJ Report No. 228416). U.S. Department of Justice Bureau of Justice Statistics, 2010 (http://bjs.ojp.usdoj.gov/content/pub/pdf/svjfry09.pdf).

[106] Greenberg MT Lippold MA Promoting healthy outcomes among youth with multiple risks: innovative approaches. Annu Rev Public Health 2013; 34: 253–70. 23297659 10.1146/annurev-publhealth-031811-124619 PMC4350237

[107] Dretzke J Davenport C Frew E Barlow J Stewart-Brown S Bayliss S The clinical effectiveness of different parenting programmes for children with conduct problems: a systematic review of randomised controlled trials. Child Adolesc Psychiatr Ment Health 2009; 3: 1–10. 10.1186/1753-2000-3-7 PMC2660289 19261188

[108] Lundahl B Risser H Lovejoy M A meta-analysis of parent training: moderators and follow-up effects. Clin Psychol Rev 2006; 26: 86–104. 16280191 10.1016/j.cpr.2005.07.004

[109] Knapp M McDaid D Pasonage M Mental Health Promotion and Mental Illness Prevention: The Economic Case. London School of Economics and Political Science Personal Social Services Research Unit, 2011.

[110] Goldson B Muncie J Towards a global ‘child friendly’ juvenile justice? Int J Law Crime Just 2012; 40: 47–64.

